# Recent Advances in Inflammation-Associated Epicardial Adipose Tissue for Atrial Fibrillation Patients

**DOI:** 10.31083/RCM36598

**Published:** 2025-07-22

**Authors:** Jiawei Li, Maomao Zhao, Lu Bai, Jing Zhao, Hanxiang Gao, Ming Bai

**Affiliations:** ^1^Department of Cardiology, First Hospital of Lanzhou University, 730000 Lanzhou, Gansu, China; ^2^Gansu Key Laboratory of Cardiovascular Diseases, The First Hospital of Lanzhou University, 730000 Lanzhou, Gansu, China; ^3^Gansu Clinical Medical Research Center for Cardiovascular Diseases, The First Hospital of Lanzhou University, 730000 Lanzhou, Gansu, China; ^4^The First School of Clinical Medicine, Lanzhou University, 730000 Lanzhou, Gansu, China

**Keywords:** epicardial adipose tissue (EAT), atrial fibrillation (AF), inflammation, biomarker

## Abstract

The relationship between inflammation and atrial fibrillation (AF) has recently attracted significant research interest. Epicardial adipose tissue (EAT) contributes to the pathogenesis of AF through its inflammatory, metabolic, and electrophysiological effects and may also influence AF outcomes. Inflammatory cells within EAT release key proinflammatory cytokines, including interleukin (IL)-1β and tumor necrosis factor-α (TNF-α), which promote cardiomyocyte apoptosis and fibrosis. These changes compromise cardiac electrophysiological stability and elevate the risk of arrhythmias. Moreover, increased EAT thickness and volume have been identified as critical biomarkers for AF risk, providing new insights into AF diagnosis and treatment. However, despite compelling evidence of a strong association between EAT and AF, further studies are needed to fully elucidate the mechanisms underlying the role of EAT and assess its potential as a therapeutic target. This review aimed to explore the specific mechanisms of inflammation-related EAT in AF and evaluate the clinical potential of EAT as a biomarker and therapeutic target.

## 1. Introduction

Atrial fibrillation (AF) represents a widespread and complex cardiac rhythm disorder, showing a strong correlation with advancing age [1]. Among individuals aged 45 years and above, the probability of developing AF during their lifetime is approximately one in four. Global epidemiological data indicate a 33% surge in AF cases over the past twenty years, with current estimates suggesting nearly 37.57 million affected individuals worldwide, representing about half a percent of the global population [2]. This upward trend is particularly evident in nations with higher economic development, though the rate of increase is more pronounced in regions with intermediate economic status [1, 3, 4, 5]. Emerging research has established significant connections between inflammation-related epicardial adipose tissue (EAT) and various systemic conditions, such as arterial plaque formation [6], cardiac rhythm disturbances, immune system irregularities [7], age-related changes [8], metabolic disorders, and excessive body weight [8, 9]. The development of AF involves a complex interplay of electrical conduction abnormalities, structural changes in atrial tissue, and impaired atrial muscle cell function, with inflammatory processes playing a pivotal role in these mechanisms. This analysis seeks to explore the specific pathways through which inflammatory EAT contributes to AF development and progression, potentially providing novel approaches for cardiovascular disease management and prevention.

## 2. The Role of EAT in Cardiovascular Disease

EAT is a metabolically active fat compartment primarily composed of adipocytes, vascular elements, and immune cells [10]. Pathological changes in EAT have been strongly associated with several cardiovascular diseases, including AF [11], coronary artery disease (CAD) [12] and heart failure [13]. Table 1 (Ref. [11, 12, 13, 14]) lists important research results related to EAT. The thickening of EAT has been recognized as an independent risk factor for CAD, with evidence showing that EAT thickness correlates with the degree of coronary stenosis, particularly in individuals with obesity and metabolic syndrome [15, 16]. Additionally, in chronic heart failure, both inflammatory responses and dysregulated fat metabolism in EAT contribute to an increased cardiac burden. The pathological alterations in EAT, characterized by the dynamic increase in both adipocytes and inflammatory cells, can impair cardiac function, further exacerbating the symptoms of heart failure [17]. Elevated EAT levels are frequently linked to the onset of cardiovascular conditions such as CAD, heart failure, and disturbances in lipid metabolism [16, 17].

**Table 1.** **The role of EAT in cardiovascular disease**.

Author (year)	Research topic	Study design	Sample size	Image/measurement method	Main finding	Evidence grade	*Refs.*
Mazurek *et al*. (2003)	“Human epicardial adipose tissue is a source of inflammatory mediators”	Laboratory investigation	45	Surgical sampling + biochemical analysis	EAT secretes pro-inflammatory cytokines (such as IL-6, TNF-α) that promote atherosclerosis.	2B	[14]
Thanassoulis *et al*. (2010)	“Pericardial fat is associated with prevalent atrial fibrillation”	Cross-sectional study	3201	CT	Each 1 standard deviation (SD) increase in pericardial fat volume was associated with a 28% higher risk of atrial fibrillation (OR = 1.28, *p* = 0.01), independent of BMI.	2A	[11]
Mahabadi *et al*. (2013)	“Association of epicardial fat with cardiovascular risk factors and incident myocardial infarction”	Prospective cohort study	4093	CT was used to measure pericardial fat thickness	For every 1 SD increase in EFV, the risk of coronary artery calcification score (CACS) increased by 18% (*p * < 0.001), and was still significant after adjusting for BMI, lipid profile, etc.	1B	[12]
Rabkin SW (2017)	“Is reduction in coronary blood flow the mechanism by which epicardial fat produces left ventricular diastolic dysfunction?”	Clinical cross-sectional study	100–150	Cardiac CT/MRI (volume) or ultrasound (thickness)	Through thickening, compression and secretion of vasoactive substances, EAT leads to decreased coronary microvascular blood flow reserve, which leads to limited left ventricular diastolic function.	2A	[13]

Abbreviations: EAT, epicardial adipose tissue; MRI, magnetic resonance imaging; EFV, epicardial fat volume; Refs, references; IL, interleukin; TNF-α, tumor necrosis factor-α; CT, computed tomography; BMI, body mass index.

### 2.1 EAT and the Onset of AF

In recent decades, there have been numerous studies on atrial fibrillation and EAT, and Table 2 (Ref. [18, 19, 20, 21, 22, 23, 24]) lists the key studies in this regard. The volume and thickness of EAT are strongly linked to the onset and severity of AF. Increased EAT thickness is a major risk factor for AF, as its excessive accumulation leads to atrial structural remodeling, which promotes AF. EAT contributes to AF development by secreting inflammatory cytokines, which can induce myocardial cell inflammation and apoptosis [25, 26]. Specifically, inflammatory mediators in EAT can increase the duration of myocardial action potential and resting membrane potential, destabilizing the heart’s electrical activity and enhancing AF susceptibility. Electrophysiological remodeling is a key mechanism in AF involving structural and functional changes in atrial cells. Research has shown that the distribution of high dominant frequency (DF) regions in AF patients correlates with EAT volume, suggesting that EAT contributes to AF through inflammation and electrical remodeling [27, 28]. Changes in DF reflect this remodeling process, particularly in persistent AF. High DF regions are often hotspots for electrophysiological remodeling, and their electrical activity characteristics can be identified using DF mapping techniques. The stability of DF is closely linked to AF persistence, with stable DF typically indicating stable atrial electrical activity sources [29]. Notably, the volume of EAT and levels of inflammatory biomarkers are higher in patients with persistent AF compared to those with paroxysmal AF, indicating that EAT may play different roles at various stages of AF [30].

**Table 2.** **The role of EAT in AF**.

Author (year)	Research topic	Study design	Sample size	Image/measurement method	Main finding	*Refs*.
Wong *et al*. (2011)	Pericardial fat is associated with atrial fibrillation severity and ablation outcome	Prospective cohort study (case-control)	102 AF patients and 20 controls	Cardiac MRI quantified EAT volume	EAT volume was independently correlated with the presence, severity, ablation and recurrence of AF.	[18]
Friedman *et al*. (2014)	Pericardial fat is associated with atrial conduction: the Framingham Heart Study	Cross-sectional study	1946 cases	CT was used to measure pericardial fat thickness	EAT thickness was positively correlated with P-wave duration (*p * < 0.002), suggesting atrial conduction delay.	[19]
Nalliah *et al*. (2020)	Epicardial adipose tissue accumulation confers atrial conduction abnormality	Cross-sectional studies combined with *in vitro* cell experiments	19 cases	CT+ electrophysiological mapping + histological analysis + cell experiment + proteomics	The volume of EpAT in the anterior wall of the right atrium was significantly correlated with slower conduction and increased electrical signal complexity, but the EAT in both atria had no such correlation.	[20]
Lin *et al*. (2012)	Adipocytes modulate the electrophysiology of atrial myocytes	Laboratory investigation	Not specified	*In vitro* electrophysiological recording + fluorescence microarray technique	The low levels of inflammatory cytokines in the supernatant of epicardial adipocytes cultured alone suggest that the interaction between adipocytes and cardiomyocytes may be realized through paracrine mechanism.	[21]
Shaihov-Teper *et al*. (2021)	Extracellular vesicles from epicardial fat facilitate atrial fibrillation	Prospective observational studies combined with *in vitro* experiments and animal model validation	Epicardial fat (eFat) samples were collected from 62 patients	Extracellular vesicles (EVs) isolation + electrophysiological experiment	The eFat in AF patients secreted more EVs, and was rich in pro-inflammatory (IL-1α, IL-6, TNF-α), pro-fibrosis (TGF-β) and pro-arrhythmia molecules. The proteome and microRNA profiles of EVs are unique in AF patients.	[22]
Haemers *et al*. (2017)	Atrial fibrillation is associated with the fibrotic remodelling of adipose tissue in the subepicardium of human and sheep atria	Observational study combined with animal model experiments	92 cases (human) + unspecified number (animal)	Histological analysis + imaging examination + inflammation analysis	The degree of subepicardial fat infiltration was higher in patients with permanent atrial fibrillation. AF sheep showed dense fiber-fat infiltration in the left atrium, consistent with human samples.	[23]
De Coster *et al*. (2018)	Arrhythmogenicity of fibro-fatty infiltrations	Computer simulation research	Not specified	Mathematical model	Adipose infiltration is more likely to induce arrhythmia than fibrosis alone (30% non-conductive tissue is required).	[24]

TGF-β, transforming growth factor-beta; AF, atrial fibrillation.

### 2.2 EAT and AF Prognosis

Recent studies have highlighted the significant association between EAT and both the onset and prognosis of AF. EAT enlargement may contribute to AF through multiple mechanisms. Specifically, adipocytes within EAT secrete pro-inflammatory cytokines, which lead to chronic inflammation in cardiac tissue, resulting in myocardial fibrosis and electrophysiological changes that promote both the initiation and persistence of AF [25, 30]. Moreover, free fatty acids released from EAT may adversely affect cardiac electrophysiology by increasing myocardial cell excitability, thereby triggering AF [26]. EAT’s metabolic activity is tightly connected to heart function, and metabolic abnormalities may predispose the heart to AF [31]. Several studies have shown that EAT volume is significantly associated with AF recurrence. For example, patients with EAT volumes greater than 92 cm^3^ who undergo catheter ablation are nearly twice as likely to experience AF recurrence compared to those with smaller EAT volumes [32]. Furthermore, the EAT volume surrounding the atria is especially linked to AF recurrence, with larger volumes of EAT decreasing the likelihood of maintaining sinus rhythm [33, 34]. These findings suggest that EAT plays a pivotal role in AF recurrence. EAT volume and its metabolic activity may serve as valuable prognostic markers for AF patients. Increased EAT volume has also been shown to correlate with a higher risk of cardiovascular events in AF patients [35, 36]. Additionally, EAT changes may predict treatment success; for example, a significant reduction in EAT volume following catheter ablation could indicate a favorable response to treatment and a lower risk of recurrence. Tracking EAT changes provides an opportunity for clinicians to adjust treatment plans based on individual patient needs, potentially improving treatment outcomes.

## 3. How Inflammation Affects Atrial Fibrillation

### 3.1 The Role of Inflammatory Markers

Inflammatory cytokines are pivotal in initiating atrial electrophysiological and structural changes predisposing individuals to AF [37, 38]. Serum levels of inflammatory markers, including C-reactive protein (CRP), tumor necrosis factor-α (TNF-α), and interleukin (IL)-6, correlate strongly with AF’s prevalence, duration, and clinical outcomes [39, 40, 41]. Elevated CRP levels are particularly indicative of AF’s chronicity, highlighting systemic inflammation’s critical role in its perpetuation. In AF patients, pronounced inflammatory cell infiltration within atrial tissues can lead to significant structural alterations and impair electrical signal propagation, facilitating AF onset [37, 42]. Additionally, inflammatory responses to pleural effusions post-cardiac surgery, manifesting as pericarditis, are associated with increased AF risk by disturbing cardiac electrophysiology [43, 44, 45]. The study by Racca *et al*. [46] is the first to systematically investigate the dynamic changes of multiple cytokines in the serum of patients after cardiac surgery, especially during rehabilitation. The results showed that a disintegrin and metalloproteinase 17 (ADAM17) and IL-25 levels were significantly associated with the occurrence of postoperative atrial fibrillation (POAF). Specifically, IL-25 levels above 4 pg/mL may be protective against POAF, while ADAM17 levels below 47.3 pg/mL may be a potential risk factor for the development of POAF. These findings suggest that ADAM17 and IL-25 may be used as novel biomarkers to predict POAF, providing an important basis for early clinical identification of high-risk patients. In addition, the study also revealed the positive regulatory effect of cardiac rehabilitation on inflammatory markers, suggesting that rehabilitation intervention may improve patient prognosis by reducing postoperative inflammatory response [46]. In patients with autoimmune diseases, the incidence of AF is notably higher, and the systemic inflammatory response is considered one of the potential triggers of AF. These patients often exhibit extensive myocardial electrical instability, and chronic systemic inflammation might promote arrhythmias through indirect pathways (such as accelerating the occurrence of ischemic heart disease and congestive heart failure) or by directly affecting cardiac electrophysiology [47, 48, 49]. These phenomena indicate that systemic inflammatory responses may promote the occurrence of AF through various mechanisms in multiple diseases. Therefore, assessing inflammatory markers in EAT is vital for elucidating its contribution to AF and other cardiovascular conditions. Current methods for evaluating these markers include tissue biopsy, gene expression analysis, and biochemical assays. By acquiring EAT biopsy samples, researchers can examine both cellular composition and the expression levels of key inflammatory markers. Techniques like RNA extraction are commonly used to assess the expression of specific inflammatory genes, providing valuable insights into the mechanisms underlying cardiovascular diseases [50]. Modulating the activity of these markers may offer novel strategies to improve patient prognosis and reduce the risk of cardiovascular events.

### 3.2 Atrial Remodeling

Atrial remodeling is a fundamental mechanism underlying AF, involving electrophysiological disturbances and structural alterations in the atria. Inflammatory mediators activate atrial fibroblasts, promoting fibrosis. Atrial fibrosis further impairs atrial electrical conduction, creating a vicious cycle that increases the risk of AF [51, 52]. Myocardial fibrosis, characterized by increased collagen deposition in the myocardial interstitium, leads to significant structural and functional changes. This fibrotic process is closely regulated by pro-fibrotic factors such as transforming growth factor-beta (TGF-β) and chronic inflammation, which drive fibroblast proliferation and collagen synthesis. Chronic inflammation can also cause myocardial cell damage, further accelerating the fibrosis process. Inflammatory factors EAT involved in the occurrence and progression of myocardial fibrosis through multiple signaling pathways. Inflammatory cytokines such as TNF-α and IL-6 secreted by EAT activate inflammatory cells in the myocardium, leading to myocardial cell apoptosis and damage. This leads to the activation of myocardial fibroblasts, enhanced collagen synthesis, and fibrosis [53, 54]. Additionally, cytokines such as TGF-β and IL-6, secreted by EAT, upregulate the expression of fibrotic factors in myocardial cells, thereby promoting fibrosis [55, 56, 57]. Inflammatory factors in EAT not only affect myocardial cells but also alter the heart’s electrophysiological properties and contractile function, potentially leading to reduced cardiac compliance and complications such as heart failure [58]. Thus, targeting inflammation in EAT could be a promising strategy for treating myocardial fibrosis and mitigating AF progression.

### 3.3 Relationship Between Inflammation, Neuroendocrine, and Immune Modulation in EAT

EAT exhibits complex interactions between its neuroendocrine, immune, and inflammatory responses, which influence arrhythmia development through multiple mechanisms. These interactions influence arrhythmogenesis through multiple pathways, including changes in cardiac electrophysiological properties, chronic myocardial damage mediated by inflammation, and neuroregulation. Bioactive substances secreted by EAT, such as leptin, directly affect myocardial cell membrane potentials and action potential duration, which increases arrhythmia risk [30, 59]. Chronic inflammation in EAT raises cytokine levels, inducing myocardial apoptosis and fibrosis, destabilizing cardiac electrical activity, and promoting arrhythmias. Furthermore, the neuroendocrine function of EAT affects autonomic nervous system balance, with sympathetic activation and parasympathetic inhibition leading to irregular heart rate and increased arrhythmia risk. Metabolic dysfunction, often seen in obesity, disrupts EAT function, exacerbating inflammation and perpetuating a cycle that increases arrhythmia risk. The interplay between the neuroendocrine, immune, and inflammatory functions of EAT forms a complex network that collectively impacts heart health. Further investigation into these mechanisms will provide essential insights for developing novel therapies for arrhythmias and offer new perspectives in managing cardiovascular diseases.

## 4. Mechanisms Linking Inflammation to Atrial Fibrillation

### 4.1 The Role of Inflammatory Markers

Inflammatory cytokines play a central role in driving atrial electrophysiological and structural remodeling, which predisposes individuals to AF [37, 38]. Elevated serum levels of inflammatory markers, including CRP, TNF-α, and IL-6, are strongly correlated with AF prevalence, persistence, and adverse clinical outcomes [39, 40, 41]. CRP elevation, in particular, serves as a biomarker of chronic AF, reflecting systemic inflammation’s role in sustaining arrhythmia. Histopathological studies reveal inflammatory cell infiltration in atrial tissues, contributing to structural damage and disrupted electrical conduction—key mechanisms in AF initiation and perpetuation [37, 42]. Post-cardiac surgery inflammation, such as pericarditis secondary to pleural effusions, further increases AF risk by altering cardiac electrophysiology [43, 44, 45]. Additionally, patients with autoimmune disorders exhibit higher AF incidence, likely due to systemic inflammation promoting myocardial electrical instability through direct electrophysiological effects or indirect pathways (e.g., ischemic heart disease, heart failure) [47, 48, 49]. These observations underscore the multifaceted role of inflammation in AF pathogenesis across diverse clinical contexts.

Current research emphasizes the assessment of inflammatory markers in EAT, a metabolically active fat depot surrounding the heart. Techniques such as tissue biopsy, gene expression profiling, and biochemical assays enable the evaluation of EAT’s cellular composition and inflammatory mediator expression (e.g., RNA analysis of pro-inflammatory genes) [50]. Targeting these inflammatory pathways may offer novel therapeutic strategies to mitigate AF progression and improve cardiovascular outcomes.

### 4.2 Inflammatory-Driven Atrial Remodeling

Atrial remodeling—encompassing electrophysiological dysfunction and structural fibrosis—is a hallmark of AF pathophysiology. Inflammatory mediators activate atrial fibroblasts, triggering collagen deposition and interstitial fibrosis. This fibrotic process disrupts electrical signal propagation, creating a self-perpetuating cycle that sustains AF [51, 52]. Key pro-fibrotic factors such as TGF-β and chronic inflammatory states drive fibroblast proliferation and collagen synthesis [53, 54]. Myocardial injury caused by persistent inflammation further accelerates fibrosis. EAT contributes to this process through paracrine signaling. Inflammatory cytokines (e.g., TNF-α, IL-6) secreted by EAT induce myocardial apoptosis, fibroblast activation, and collagen overproduction. Concurrently, EAT-derived TGF-β and IL-6 upregulate fibrotic gene expression in cardiomyocytes, exacerbating structural remodeling [55, 56, 57]. Beyond fibrosis, these inflammatory mediators alter cardiac electrophysiology and contractility, potentially leading to reduced ventricular compliance and heart failure [58]. Thus, modulating EAT-associated inflammation represents a promising therapeutic target to disrupt fibrotic progression and AF recurrence.

### 4.3 Interplay of Inflammation, Neuroendocrine, and Immune Pathways in EAT

EAT functions as a neuroendocrine-immune organ, with its inflammatory activity intricately linked to arrhythmogenesis. Bioactive molecules secreted by EAT, including leptin, directly influence cardiomyocyte membrane potentials and action potential duration, increasing susceptibility to arrhythmias [30, 59]. Chronic inflammation within EAT elevates systemic cytokine levels, promoting myocardial apoptosis, fibrosis, and electrical instability.

EAT also interacts with the autonomic nervous system (ANS): sympathetic hyperactivity and parasympathetic suppression—mediated by EAT-derived neuroendocrine factors—predispose to heart rate variability and arrhythmia. In obesity-related metabolic dysfunction, EAT expansion exacerbates inflammation, creating a vicious cycle that amplifies arrhythmia risk. This triad of neuroendocrine, immune, and inflammatory mechanisms in EAT underscores its role as a critical modulator of cardiac electrophysiology and structural integrity.

The synergistic effects of inflammation, atrial remodeling, and EAT-mediated pathways form a complex network driving AF pathogenesis. Elucidating these interactions—particularly the cross-talk between inflammatory markers, fibrotic pathways, and neuroendocrine-immune regulation—holds significant translational potential. Future therapies targeting EAT inflammation or its downstream mediators may improve AF management and reduce cardiovascular morbidity.

## 5. Description of Inflammatory Signaling Pathways

**Fig. 1.**
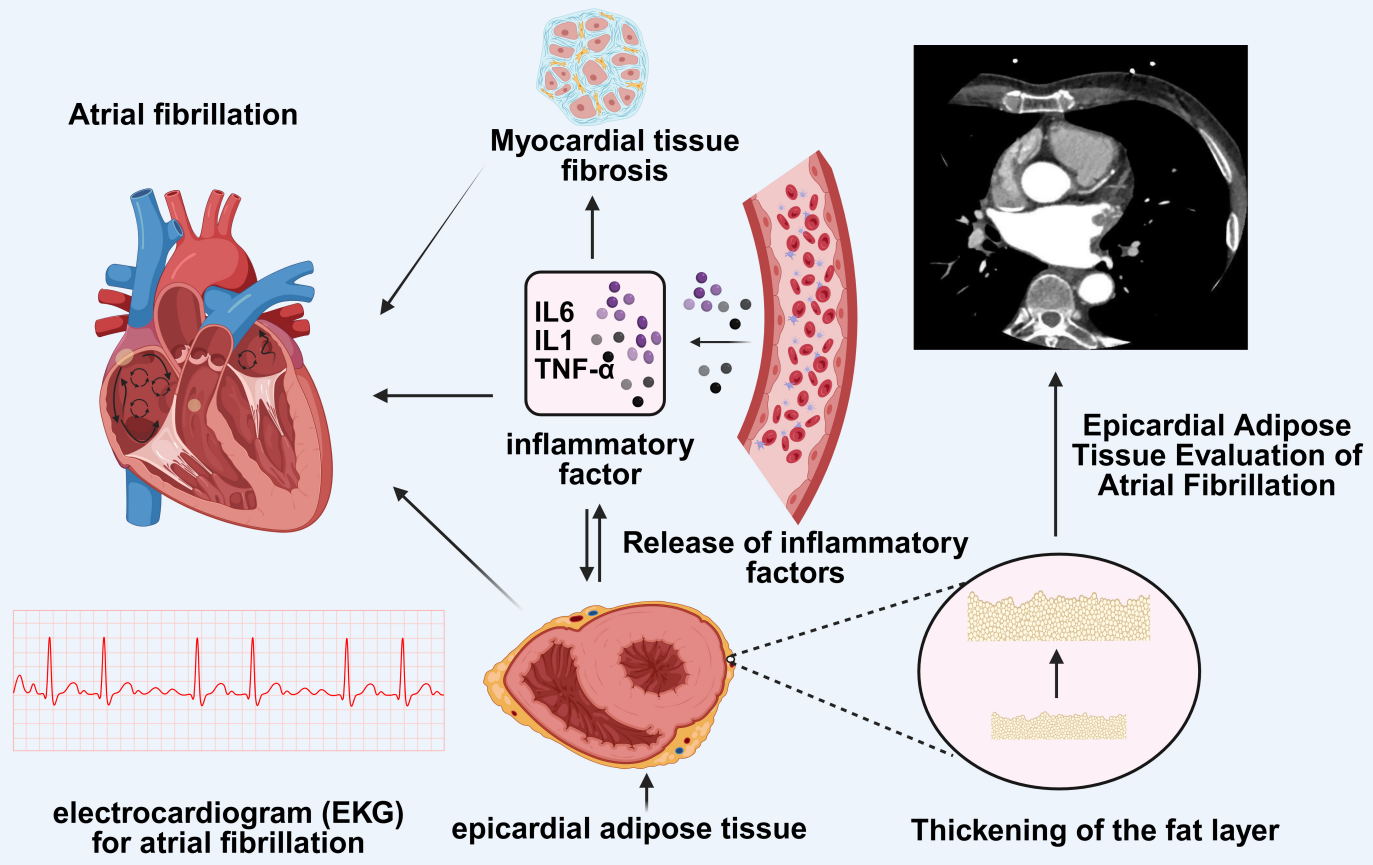
**Imaging features of EAT and the impact of inflammation in EAT on atrial fibrillation**. AF is a common arrhythmia associated with multiple factors. The figure depicts the role of EAT in AF. The imaging features of EAT can be evaluated using computed tomography or cardiac magnetic resonance imaging, which reveals the thickening of the adipose layer. EAT releases inflammatory cytokines such as IL-6, IL-1, and TNF-α, which are associated with myocardial fibrosis. Myocardial fibrosis is a key pathological basis for AF, potentially leading to alterations in cardiac electrophysiological properties, thereby promoting the onset of atrial fibrillation. Moreover, the inflammatory cytokines in EAT may directly affect cardiac electrical activity, contributing to the characteristic electrocardiographic patterns seen in atrial fibrillation. Figure created using BioRender.com.

Inflammatory signaling pathways are key mechanisms by which EAT contributes to the development of AF (Fig. 1). EAT secretes several pro-inflammatory cytokines, including TNF-α, IL-6, IL-8, and IL-1β, which initiate adjacent inflammation in the myocardium through endocrine and paracrine signaling [60] (Table 1). Immune cells in EAT, such as macrophages, stromal cells, and lymphocytes, also participate in the secretion of inflammatory mediators and chemokines, exacerbating the inflammatory milieu. These cytokines can alter the electrical activity of atrial myocytes, resulting in arrhythmias [61, 62], and may further accelerate the progression of atherosclerosis, contributing to the increased risk of AF [62, 63]. The secretion of TGF-β1, matrix metalloproteinase (MMP)2, and MMP7 by EAT is critical in promoting collagen deposition and fibrosis in the atrial tissue, contributing to atrial remodeling. Activin A enhances the expression of TGF-β1, thus stimulating the production of MMP2 and MMP7, which are involved in extracellular matrix remodeling, leading to fibrosis through the Smad signaling pathway [64, 65, 66, 67]. Additionally, EAT is a major source of reactive oxygen species (ROS), contributing to oxidative stress in AF patients [68]. Elevated ROS levels promote oxidative damage in the atrial tissue and activate kinases such as Ca^2+^/calmodulin-dependent protein kinase-II (CaMKII), further exacerbating AF [69, 70, 71]. The lipid infiltration pathway, where free fatty acids from EAT infiltrate the myocardium, leads to electrophysiological disturbances, including slowed conduction and myocardial cell disorganization, promoting reentry [30, 72, 73]. EAT is rich in autonomic nerve plexuses, where adrenergic activation increases Ca^2+^ influx, leading to afterdepolarizations and promoting AF, while cholinergic activation shortens action potential duration, facilitating reentry [74, 75, 76]. Sympathetic activation exacerbates AF by modulating myocardial metabolism, whereas parasympathetic activity may offer protective effects, although excessive parasympathetic tone may impair cardiac conduction and function [77]. In the renin-angiotensin-aldosterone system (RAAS) pathway, EAT activates Ang II signaling, which stimulates fibroblast proliferation and collagen synthesis, driving myocardial fibrosis and remodeling [78, 79, 80]. The pro-fibrotic effects of Ang II, mediated by TGF-β1, upregulate specific gene expression through the Smad signaling pathway, promoting aldosterone production and nicotinamide adenine dinucleotide phosphate (NADPH) oxidase activation, which accelerate inflammation and cell apoptosis [80, 81]. Furthermore, the nuclear factor-κB (NF-κB) signaling pathway plays a pivotal role in EAT’s chronic inflammatory response, fibrosis, oxidative stress, and autonomic dysfunction. It is activated through lipopolysaccharide (LPS) induction and Toll-like receptor (TLR) signaling, leading to the upregulation of cytokines such as IL-6, IL-1, and TNF-α [59, 82].

The Janus tyrosine kinase (JAK)-signal transducer and activator of the transcription 3 (STAT3) signaling pathway plays a pivotal role in the heart’s inflammatory response, particularly through the action of IL-6, which activates JAK-STAT3 and triggers an inflammatory cascade that downregulates gap junction proteins in cardiac tissue. This process leads to electrical remodeling of the atrium, contributing to the onset of AF [83, 84]. Moreover, the activation of the Smad signaling pathway is closely linked to both inflammation and fibrosis in cardiac pathology. Upregulation of inflammatory processes commonly accompanies fibrosis in the heart, and excessive activation of the TGF-β/Smad pathway promotes abnormal proliferation of cardiac cells and fibrosis, which in turn disrupts the heart’s electrophysiological properties. This cascade not only leads to ventricular remodeling but also increases the susceptibility to arrhythmias such as AF. Furthermore, there is critical crosstalk between the Smad and NF-κB signaling pathways. Evidence indicates that inhibiting NF-κB can enhance TGF-β/Smad signaling, reducing the inflammatory responses associated with macrophages [85]. This interaction illustrates the multifaceted role of Smad signaling in regulating both inflammation and fibrosis. Taken together, the NF-κB, JAK-STAT3, and Smad pathways are central to the pathophysiology of EAT, driving both fibrosis and inflammation and potentially altering the heart’s electrophysiological characteristics and function, thus fostering the development of AF [86, 87]. Targeting the activation of these pathways may offer novel therapeutic strategies for AF. The interplay between inflammatory and fibrotic pathways in EAT represents a complex biological process where EAT-induced fibrosis exacerbates inflammation and vice versa. Therefore, investigating this intricate relationship could lead to the development of new treatments aimed at improving the prognosis of patients with cardiovascular disease.

Inflammatory signaling pathways play a central role in the contribution of EAT to the development of AF [60] (Fig. 1) (Table 3, Ref. [30, 59, 60, 64, 65, 66, 67, 69, 70, 71, 72, 73, 74, 75, 76, 80, 81, 82, 83, 84, 85, 86, 87]). Among these, the JAK-STAT3, TGF-β/Smad, and NF-κB pathways are particularly critical in driving inflammation, fibrosis, and electrophysiological remodeling in the atrial tissue [83, 84, 86, 87]. The JAK-STAT3 signaling pathway is activated primarily by the pro-inflammatory cytokine IL-6, which is secreted by EAT [83, 84]. IL-6 binding to its receptor triggers the phosphorylation of JAK, leading to the activation of STAT3. This cascade results in the downregulation of gap junction proteins in cardiac tissue, contributing to electrical remodeling and the onset of AF [83, 84]. Additionally, the JAK-STAT3 pathway amplifies the inflammatory response by promoting the expression of other cytokines, further exacerbating atrial dysfunction [83, 84]. The TGF-β/Smad signaling pathway is another key player in EAT-mediated atrial pathology. EAT secretes TGF-β1, which activates the Smad pathway, leading to the upregulation of MMP2 and MMP7 [64, 65, 66, 67]. These matrix metalloproteinases are critical for extracellular matrix remodeling, promoting collagen deposition and fibrosis in the atrial tissue [64, 65, 66, 67]. Fibrosis disrupts the heart’s electrophysiological properties, increasing susceptibility to arrhythmias such as AF [64, 65, 66, 67]. Furthermore, Activin A, also secreted by EAT, enhances TGF-β1 expression, further amplifying fibrotic processes [64, 65, 66, 67]. The Smad pathway also interacts with the NF-κB pathway, where inhibition of NF-κB can enhance TGF-β/Smad signaling, reducing inflammatory responses mediated by macrophages [85]. The NF-κB signaling pathway is a major driver of chronic inflammation in EAT [59, 82]. It is activated through lipopolysaccharide induction and TLR signaling, leading to the upregulation of pro-inflammatory cytokines such as TNF-α, IL-6, IL-1β, and IL-8 [59, 82]. These cytokines initiate and sustain inflammation in the adjacent myocardium through endocrine and paracrine signaling [60]. NF-κB activation also contributes to oxidative stress by increasing the production of ROS, which further damages atrial tissue and exacerbates AF [68, 69, 70, 71]. ROS activates kinases such as CaMKII, leading to electrophysiological disturbances and promoting arrhythmias [69, 70, 71].

**Table 3.** **Signaling pathway mechanism related to myocardial inflammation and fibrosis**.

Signaling pathways/routes	Involved factors/molecules	Mechanisms of action	Effects	*Refs.*
Inflammatory response pathway	TNF-α, IL-6, IL-8 and IL-1β	Endocrine and paracrine actions trigger myocardial inflammation	Affect the electrical activity of atrial myocytes, resulting in irregular heart rhythm; accelerate arteriosclerosis and increase the risk of atrial fibrillation.	[60]
Atrial fibrosis pathway	TGF-β1, MMP2 and MMP7	Promote atrial collagen deposition and fibrosis	Myocardial extracellular matrix remodeling; collagen deposition and atrial structural changes.	[64, 65, 66, 67]
Oxidative stress pathway	ROS and CaMKII	Enhance kinase activity, oxidize ion channels	The formation of a higher oxidative stress state increases the production of ROS and reduces the level of antioxidants to aggravate the oxidative damage of the atrium.	[69, 70, 71]
Lipid infiltration pathway	Free fatty acids	Cause electrophysiological changes, myocardial structural disorder	The conduction of cardiomyocytes is slowed down, the lateral cell connection is lost and the myocardial structure is disturbed, causing conduction delay and reentry.	[30, 72, 73]
Autonomic nervous pathway	Adrenergic and cholinergic	Change cell membrane potential, cause electrophysiological change	The activity of the sympathetic nerve affects the electrical conductivity of the heart and the metabolism of the outer adipose tissue, which aggravates atrial fibrillation, while the activation of the parasympathetic nerve can help maintain the stability of the heart and reduce the occurrence of atrial fibrillation.	[74, 75, 76]
RAAS pathway	Ang II, TGF-β1, Smad signaling	Cause myocardial fibrosis, upregulate gene expression	By activating RAAS to produce high levels of Ang II, EAT stimulates fibroblast proliferation and collagen synthesis, leading to remodeling and fibrosis of myocardial tissue.	[80, 81]
NF-κB signaling pathway	NF-κB (p65, p50), TNF-α, TLRs, IL-6 and IL-1	Mediate NF-κB translocation, upregulate inflammatory factor expression	Overactivation of NF-κB in EAT may exacerbate the electrophysiological instability of the heart, thereby increasing the risk of AF.	[59, 82, 85, 86, 87]
JAK-STAT3 pathway	JAK, STAT3, IL-6, TGF-β and SOCS	Trigger inflammatory cascade, downregulate gap junction proteins	IL-6 produced by EAT triggers an inflammatory cascade, down-regulates cardiac gap connexin, leads to atrial electrical remodeling, and induces AF.	[83, 84, 86, 87]
Smad signaling pathway	TGF-β, Smad2-4, CTGF and PAI-1	Smad signaling pathway	If the TGF-β level in EAT is increased, the Smad signaling pathway is activated, and then atrial fibrosis is promoted, leading to the remodeling of atrial structure, which interferes with electrical signal conduction and increases the incidence of atrial fibrillation.	[85, 86, 87]

RAAS, renin-angiotensin-aldosterone system; MMP, matrix metalloproteinase; ROS, reactive oxygen species; CaMKII, Ca^2+^/calmodulin-dependent protein kinase-II; Ang II, angiotensin II; NF-κB (p65, p50), nuclear factor-κB (p65, p50); TLRs, Toll-like receptors; JAK, Janus tyrosine kinase; STAT3, signal transducer and activator of the transcription 3; SOCS, suppressor of cytokine signaling; CTGF, connective tissue growth factor; PAI-1, plasminogen activator inhibitor-1.

In addition to these pathways, individual cytokines and mediators secreted by EAT play distinct roles in AF pathogenesis. TNF-α and IL-1β are potent pro-inflammatory cytokines that alter the electrical activity of atrial myocytes, leading to arrhythmias [61, 62]. IL-6 not only activates the JAK-STAT3 pathway but also contributes to systemic inflammation and atrial remodeling [83, 84]. IL-8 acts as a chemokine, recruiting immune cells such as macrophages and lymphocytes to the atrial tissue, further amplifying the inflammatory milieu [60]. MMP2 and MMP7, regulated by the TGF-β/Smad pathway, are critical for extracellular matrix remodeling and fibrosis, which disrupts atrial conduction and promotes reentry mechanisms [64, 65, 66, 67]. ROS, generated in excess by EAT, induces oxidative damage, activates pro-fibrotic signaling, and exacerbates electrophysiological disturbances [68, 69, 70, 71].

The interplay between these signaling pathways and cytokines creates a vicious cycle of inflammation and fibrosis in EAT, which drives atrial remodeling and increases the risk of AF [86, 87]. Targeting these pathways, such as inhibiting NF-κB, modulating JAK-STAT3, or suppressing TGF-β/Smad signaling, may offer novel therapeutic strategies for AF [86, 87]. Understanding the complex interactions between these pathways and individual cytokines could pave the way for innovative treatments aimed at improving outcomes for patients with cardiovascular disease.

## 6. Metabolic Changes of EAT During Inflammation

The metabolic alterations in EAT during inflammatory states are complex, involving changes in adipocyte function, immune cell infiltration, and the activation of various metabolic signaling pathways. Chronic low-grade inflammation disrupts the metabolic function of EAT, which in turn increases the risk of conditions such as insulin resistance and metabolic syndrome. In the context of AF-associated inflammation, EAT is a specialized form of fat storage that influences cardiac health and disease. Particularly, inflammatory changes in EAT are critical in modulating cardiac function. Under physiological conditions, EAT helps regulate the toxic concentration of fatty acids between the myocardium and the surrounding vasculature while secreting anti-inflammatory and anti-fibrotic cytokines to exert protective effects [68]. However, during pathological inflammation, the quantity and diversity of inflammatory cells in EAT increase, which reflects localized inflammatory responses [69]. Inflammation within the heart is a well-established contributor to arrhythmogenesis, and the thickening of EAT can alter the electrophysiological properties of the heart and promote structural remodeling, thereby increasing the risk of arrhythmias such as AF [70, 71].

### 6.1 Inflammatory Cell Infiltration

EAT normally contains a controlled number of inflammatory cells that contribute to immune homeostasis and tissue repair. In inflammatory conditions, however, there is a significant increase in the infiltration of inflammatory cells, particularly macrophages and T lymphocytes. This accumulation exacerbates local inflammatory responses and releases pro-inflammatory cytokines such as TNF-α and IL-6 [59]. The spatial distribution of these inflammatory cells within EAT varies by cell type. Macrophages are predominantly located around adipocytes, forming an “infiltrative” pattern, whereas lymphocytes are more likely to accumulate in the peripheral regions of the tissue. This distribution is closely associated with the extent and duration of inflammation. In localized inflammatory states, the infiltration is typically confined to the affected areas, reflecting a targeted immune response. In contrast, during systemic inflammation (such as obesity or metabolic syndrome), inflammatory cells in EAT are more diffusely distributed, contributing to a more widespread inflammatory response. In addition to local inflammation, activated immune cells in EAT release various cytokines and chemokines, which can not only exacerbate the local inflammatory environment but may also influence the function of distant organs [14, 88]. Inflammatory states can also induce apoptosis in EAT, altering its cellular composition and function and thereby worsening the pathological condition of the heart [89]. In conclusion, the infiltration of inflammatory cells in EAT displays complex and dynamic changes during inflammation, influenced by the inflammatory response’s type, duration, and systemic immune status. These changes have profound implications for both EAT function and cardiovascular pathology. Thus, understanding these mechanisms is critical for developing targeted therapeutic strategies for cardiovascular diseases.

### 6.2 Inflammatory-induced Morphological Changes in EAT Adipocytes

Adipocytes in EAT undergo a series of morphological alterations under inflammatory conditions, which not only affect their size and shape. Still, they may also have far-reaching consequences on their function, thereby influencing metabolic health. Adipocyte hypertrophy, characterized by increased cell size due to lipid accumulation, is a common feature of EAT during chronic inflammation, especially in obese individuals. In obesity, hypertrophy leads to the accumulation of lipid droplets within adipocytes, which alters their shape and structure [90]. Moreover, inflammation may lead to changes in adipocyte numbers, often through hyperplasia, the formation of new adipocytes from mesenchymal stem cells (MSCs). Certain signaling pathways are activated during inflammation, promoting this transformation and forming more mature adipocytes [90]. Interestingly, adipocytes in inflammatory environments may exhibit irregular shapes, departing from the typical round or oval morphology. These morphological alterations are closely associated with uneven lipid distribution, cell membrane changes, and cytoskeleton reorganisation, indicating adipocyte adaptive responses to inflammatory signals [91]. Multiple signaling pathways within adipocytes are reactivated during inflammation, leading to these morphological changes. For instance, pro-inflammatory cytokines such as TNF-α and IL-6 are elevated during obesity and inflammation, contributing to adipocyte hypertrophy and possibly altering their metabolic state and morphology [92, 93]. Immune cell infiltration during inflammation also releases various cytokines and chemokines, directly influencing adipocyte behavior and morphology. Macrophage infiltration, for example, can lead to changes in adipocyte morphology, thereby affecting their function. The morphological changes in adipocytes are closely linked to alterations in their metabolic functions. Under inflammation, adipocytes may experience altered metabolic pathways, affecting their lipid storage and release capacity. These changes can lead to glucose and lipid metabolism disruptions, increasing the risk of metabolic diseases. Furthermore, in inflammatory states, changes in EAT adipocytes can also influence the distribution and function of inflammatory cells. Research has shown that inflammation can induce adipocyte apoptosis and degeneration, releasing large amounts of inflammatory mediators and exacerbating local inflammation. This interaction forms a vicious cycle that promotes the accumulation of inflammatory cells in EAT, further exacerbating cardiovascular pathological processes. In conclusion, these morphological changes reflect the adaptive responses of adipocytes to environmental changes during inflammation, highlighting the complex relationship between adipocyte morphology and function in the study of obesity and related diseases.

### 6.3 Cytokine Alterations

EAT undergoes profound changes in its cytokine secretion profile during inflammatory states, a phenomenon that plays a key role in the pathogenesis of cardiovascular diseases. In particular, anti-inflammatory cytokines such as adiponectin are downregulated, while pro-inflammatory cytokines, including CRP, are upregulated. This disruption in the balance of cytokine secretion is considered a significant factor in the development and progression of cardiovascular pathologies [59, 94]. Compared to subcutaneous adipose tissue, EAT exhibits a markedly higher expression of pro-inflammatory cytokines like IL-6 and TNF-α, likely attributable to the tissue’s unique microenvironment and anatomical proximity to the heart [14, 95]. Additionally, ROS levels in EAT are elevated, while the expression of antioxidant enzymes is reduced, creating an imbalance that exacerbates oxidative stress and promotes inflammation [96]. ROS acts as a potent stimulus for cytokine release, further amplifying the inflammatory cascade within EAT. Furthermore, the inflammatory effects of EAT are not restricted to the local environment; they can propagate through the bloodstream, influencing systemic inflammation and affecting other tissues and organs. Consequently, the inflammatory processes in EAT are a complex and clinically significant area of investigation, particularly for conditions such as AF, where EAT’s inflammatory profile plays a pivotal role in cardiovascular disease progression. The cytokine profile in EAT is also modified in obesity-related metabolic disorders, such as type 2 diabetes, where the expression of resistin and leptin is significantly increased. These changes promote the production of pro-inflammatory cytokines, which contribute to metabolic disturbances such as insulin resistance [89, 97].

## 7. The Role of EAT as a Diagnostic Strategy and Therapeutic Target

The inflammatory biomarkers in EAT suggest it could be a valuable biomarker for AF. By measuring the levels of inflammatory cytokines and other markers in EAT, it is possible to assess both the risk and prognosis of AF. Additionally, EAT thickness and its inflammatory condition may offer new perspectives for personalized treatment strategies. According to research by Korantzopoulos *et al*. [52], elevated inflammatory markers in EAT are closely linked to the development and recurrence of AF, with more pronounced localized inflammation observed within the atrium. By monitoring changes in these inflammatory markers, clinicians could better predict patient outcomes in AF. Targeting EAT inflammation through interventions, such as anti-inflammatory drugs, may help lower AF incidence [98]. Despite existing studies providing initial evidence, additional clinical and basic research is needed to validate these findings further. Continued investigation may lead to the development of novel therapeutic approaches that not only reduce the burden of AF but also enhance patients’ quality of life.

### 7.1 The Role of EAT in Clinical Management

EAT demonstrates unique clinical value in the early identification and prognostic management of AF. Its core strength lies in providing dynamic information on cardiac metabolic status through non-invasive methods, offering objective evidence for clinical decision-making. Imaging techniques such as cardiac computed tomography (CT) enable precise quantification of EAT volume and spatial distribution. By establishing EAT volume thresholds, clinicians can identify high-risk populations, particularly in obese or metabolic syndrome patients, where this structural biomarker complements traditional risk assessment tools. Dynamic changes in EAT serve as a biological indicator of therapeutic response. For example, reduced EAT volume post-catheter ablation is associated with lower AF recurrence rates [30], while decreased EAT-derived inflammatory markers (e.g., IL-6) following lifestyle interventions may reflect improved cardiometabolic health [56, 99]. Furthermore, persistent EAT proliferation is closely linked to long-term AF recurrence risk [96, 99], providing a basis for personalized follow-up strategies. EAT exerts both local and systemic effects: its secreted inflammatory mediators directly promote atrial electrical remodeling and indirectly exacerbate AF risk by aggravating systemic pathologies such as hypertension and insulin resistance [100]. This dual role positions EAT as a critical nexus between arrhythmias and metabolic cardiovascular diseases, enhancing its utility in comprehensive risk stratification models.

### 7.2 Potential Impact of Inflammation-targeted Therapies on AF

Applying inflammation-targeted therapies in cardiovascular diseases has made remarkable strides in recent years. Biologic agents, particularly those inhibiting IL-1β, have demonstrated efficacy in improving cardiovascular outcomes. IL-1β antibodies, for instance, have shown promise in reducing myocardial infarction and angina in patients with atherosclerosis [9, 101]. Additionally, IL-1β receptor antagonists effectively prevent myocardial dysfunction and arrhythmias in Kawasaki disease, underscoring the potential of IL-1β-targeted treatments for arrhythmias [102]. These findings suggest that targeting IL-1β may revolutionize the management of cardiovascular diseases. Novel small molecule drugs designed to target specific inflammatory pathways have also demonstrated potential in slowing cardiovascular disease progression. These agents can suppress the release of pro-inflammatory cytokines, reducing myocardial cell damage [103, 104]. The advent of gene editing technologies further expands the possibilities for treating cardiovascular disease by directly altering inflammation-related genes, such as TNF-α, to reduce cardiac inflammation and arrhythmia risk [103]. Similarly, increasing the expression of anti-inflammatory cytokines like IL-10 may provide therapeutic benefits. Gene editing, especially when targeting ion channels and myocardial remodeling, could offer new, precise options for arrhythmia management. However, ensuring the safety and efficacy of these technologies, as well as addressing ethical concerns, will be key challenges in future research and clinical applications. Cell therapies also show promise in regulating inflammation. Introducing specific types of cells can promote cardiac repair and regeneration, improving cardiovascular function. Preclinical studies show that pluripotent stem cell-derived cardiomyocytes (PSC-CM) can regenerate damaged heart tissue, improve left ventricular ejection fraction (LVEF), and reduce fibrosis, providing strong evidence for the potential of stem cells in AF treatment [105, 106]. Several clinical trials have already validated the efficacy of inflammation-targeted therapies in AF. Non-specific anti-inflammatory agents, such as statins, have been proposed to reduce the risk of AF occurrence [107, 108, 109]. However, due to their broad mechanism of action, these agents may lack precision in targeting the core inflammatory pathways implicated in AF. In contrast, targeted anti-inflammatory therapies directed at specific inflammatory pathways (e.g., blocking IL-1, IL-6, or IL-17) have demonstrated potential antiarrhythmic effects in preclinical studies, though clinical translation requires further evidence. For instance, the CONVERT-AF pilot trial observed a trend toward reduced AF recurrence after a single dose of canakinumab (an IL-1β monoclonal antibody) post-electrical cardioversion, but its statistical significance awaits validation through larger-scale studies [110]. These findings suggest that precise cytokine-targeted interventions may offer a novel direction for AF management.

Current randomized controlled trials (RCTs) are actively investigating the efficacy of anti-inflammatory drugs in AF patients. For example, colchicine, while early studies indicated potential protective effects against postoperative AF, recent clinical trials have yielded conflicting results. The COP-AF trial demonstrated that colchicine failed to significantly reduce AF incidence after non-cardiac thoracic surgery [111], and the IMPROVE-PVI trial did not confirm its efficacy in decreasing AF recurrence post-pulmonary vein isolation [110]. Collectively, these outcomes highlight that monotherapy with non-specific anti-inflammatory agents may be insufficient to markedly improve AF outcomes, whereas targeted interventions against specific inflammatory pathways (e.g., IL-1, IL-6, or IL-17) may hold greater therapeutic promise. Building on these insights, future research may explore combined anti-inflammatory strategies to optimize therapeutic efficacy. For example, combining cytokine-targeted therapies (e.g., IL-1 inhibitors) with non-specific anti-inflammatory agents (e.g., statins) could synergistically inhibit inflammatory cascades through multi-pathway mechanisms, thereby reducing AF recurrence risk.

### 7.3 EAT-related Stem Cell Therapy

EAT disrupts cardiac electrophysiology by promoting arrhythmia through electrotonic coupling between resident stem cells and cardiomyocytes [112]. Conversely, therapeutic stem cells can mitigate this risk by normalizing electrical coupling between cells, thereby reducing EAT-related conduction delays. In addition to electrical regulation, stem cells may play a paracrine role—secreting bioactive factors that improve the pathological microenvironment of EAT and indirectly inhibit its arrhythmic activity [113]. Globally, multiple clinical trials are evaluating the safety and therapeutic efficacy of human pluripotent stem cells (hPSCs) in arrhythmia management, with a primary focus on their cardiomyogenic differentiation potential and functional cardiac repair capabilities [114]. Parallel investigative efforts are exploring combined gene-editing and stem cell therapies, where genetic modifications (e.g., enhanced gap junction protein expression) are employed to augment the antiarrhythmic properties of transplanted cells [115]. While preliminary outcomes appear promising, widespread clinical adoption of these approaches remains limited by scalability and long-term safety considerations. Notably, emerging trials target the inflammatory microenvironment of EAT, investigating stem cell-mediated immunomodulation of CD8+ T cells and other immune populations to attenuate arrhythmogenic triggers [116]. Stem cell therapy has shown great potential in inhibiting the arrhythmic effects of EAT and may provide a new strategy for the treatment of arrhythmia. However, research is still in its early stages, and more clinical trials and mechanistic studies are needed to verify its safety and efficacy.

## 8. Conclusion and Perspectives 

The relationship between EAT and AF is intricate and multifaceted. EAT contributes to the onset of AF and influences its prognosis. Thus, a detailed assessment of EAT can offer valuable information for managing AF patients. This review highlights the role of EAT in AF through inflammatory, metabolic, and electrophysiological mechanisms. We propose that routine EAT evaluation be integrated into clinical practice for AF patients to support personalized treatment plans and assess the impact of interventions aimed at reducing EAT volume on AF outcomes. While existing research has elucidated the connection between EAT and AF, further prospective studies are required to validate EAT as a biomarker and to assess its clinical applicability in AF management. These studies will contribute to a more robust mechanistic model to guide clinical decisions.
